# A micro-CT study of the pulp cavity morphology of maxillary fourth premolar teeth in dogs

**DOI:** 10.3389/fvets.2024.1499465

**Published:** 2024-11-15

**Authors:** Marie-Christine Morin, Jérôme D'Astous

**Affiliations:** Centre Vétérinaire Daubigny, Québec City, QC, Canada

**Keywords:** micro-CT, endodontic, dog, pulp cavity, maxillary fourth premolar teeth, root canal treatment (RCT)

## Abstract

**Introduction:**

The objectives of the present study were (1) to describe the anatomy of the endodontic system of the dog's maxillary fourth premolar tooth (MxPM4) in relation to the morphology of the crown, (2) to determine if variations of the endodontic system exist, and (3) to look at the implications for endodontic treatment.

**Methods:**

Ten MxPM4 were harvested en bloc and scanned using micro-computed tomography (micro-CT).

**Results:**

The morphology of the pulp chamber mostly corresponded with the shape of the crown. Three pulp horns were clearly visible and related to the paracone, the metacone, and the metastyle. Nevertheless, the pulp horns of the metacone and metastyle could be fused, partially fused or distinct. Other pulp projections were also present, but rarely, in the parastyle, the protocone, and the plesioconule. All teeth showed a noticeable angulation of an average of 150 degrees at the coronal third of the mesiopalatal canal.

**Discussion:**

Thus, the most common transcoronal approach for root canal treatment does not allow a straight access to the apex. There were also minor variations in the locations of the canal orifices. This first micro-CT study of the MxPM4 in dogs showed anatomical features and variations of the pulp cavity that have not been described before.

## Introduction

The maxillary fourth premolar tooth (MxPM4) is a major tooth in dogs because of its size and function. Together with the mandibular first molar tooth, they form the carnassial teeth, which produce powerful scissors and are the main teeth for chewing. As a result, it is not surprising that the MxPM4 is often found with complicated crown fractures that involve pulp exposure and subsequent pulp infection, pulp necrosis and apical periodontitis (1, 2). Endodontic treatment, most frequently root canal treatment, is then needed to preserve the tooth and its crucial role in mastication.

Understanding the internal anatomy of the tooth is of great importance in the success of a root canal treatment. The pulp cavity morphology could influence key steps of the procedure such as the coronal access preparation, shaping of the canals, disinfection and obturation (3). However, this knowledge is limited in veterinary dentistry, including for the MxPM4 in dogs. The pulp cavity is the inner part of the tooth that contains the pulp. It is divided into two portions: the pulp chamber within the crown and the pulp canal within the root. The pulp is vital and made of nerves, blood vessels, interstitium and dentin surface-lining odontoblasts (2, 4). The outline of the pulp generally corresponds with the external contour of the tooth (4). The pulp chamber of the MxPM4 is known to have a pulp horn (or pulp extension) that is central, and which matches the main cusp. It is usually where a fracture will occur and expose the pulp cavity. The MxPM4 has three canals which correspond to three different roots. Two are located mesially and are called mesiobuccal (B) and mesiopalatal (P). The third canal is found distally (D) (5). In dogs, the neurovascular tissues enter the apex of roots through multiple foramina called the apical delta (6). Non-apical ramifications (NAR) could also be found in some roots in 68% of the MxPM4 (7). Eisner described the position of the three canals for endodontic access in 1990 (8). According to his experience, the position of the P canal, as it emerges from the pulp chamber, is variable.

**Figure 1:**
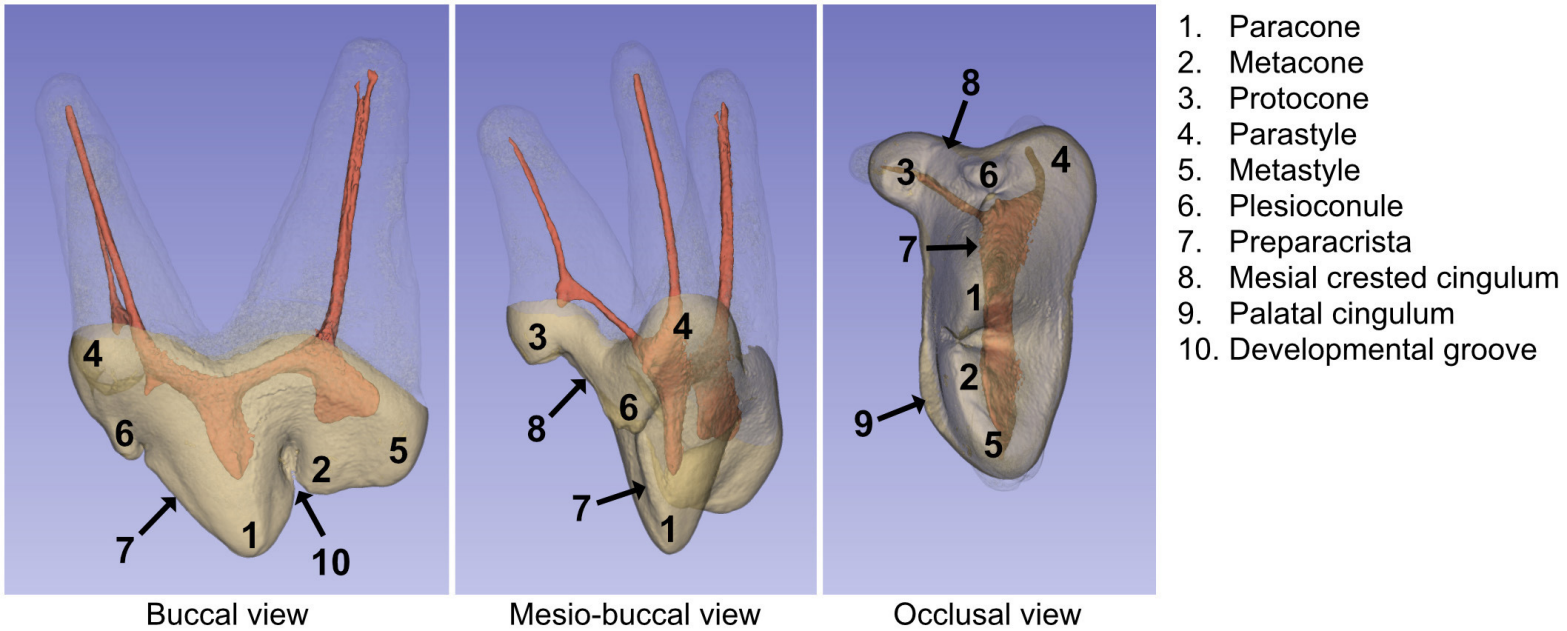
Landmarks on the crown of the maxillary fourth premolar in dogs according to the Osborn's terminology. Specimen 5–208 is shown. Note that a developmental groove is a term used in dentistry in general. The roots were segmented here to aid illustration of the whole tooth morphology.

The topography of the crown in Canidae has been described primarily in evolutionary science, where the Osborn's terminology is predominant. This nomenclature was used here to identify the outer structure of the crown (9) (Figure 1). The most prominent cusp is the paracone and is attached to the metacone, the second-largest cusp located distally. A developmental groove, a term used in dentistry, separates the two (2, 10). Together, they form two sharp triangular cusps that are buccally compressed (11). The third and last principal cusp of the tritubercular theory on tooth evolution, the protocone, is compact and is located mesiopalatally. The metacone is coalescent with the metastyle, a minor cusp and the most distal part of the crown. The parastyle is another minor prominence and is at the mesiobuccal extremity. The crest that runs from the paracone and mesially is called the preparacrista (12). The plesioconule is a minor cusp at the extremity of the preparacrista (9). The MxPM4 also has a ridge called a cingulum that is marked palatally and more discreet mesially.

Since the 90's, micro-CT has allowed great advances in human dentistry in the knowledge of endodontic morphology. This technology uses X-rays to view teeth *ex vivo* in multiple planes with sections as thin as a few micrometers (μm). The whole tooth or segmentation of different anatomical structures could then be reconstructed in three dimensions for further visualization and analysis. Few studies exist in the veterinary literature that used micro-CT to describe teeth, with only two studies applicable to small animal dentistry (13–17). The objectives of this study were (1) to describe the anatomy of the endodontic system of the dog's MxPM4 in relation to the morphology of the crown, (2) to determine if variations of the endodontic system exist, and (3) to discuss the implications for endodontic treatments.

## Materials and methods

### Dog selection and specimen collection

The MxPM4 from 10 dogs' cadaver heads of unknown breed and age were inspected. Dogs were euthanized for reasons unrelated to the study and owners donated the body for research purposes. Cadavers were kept frozen until tooth selection. The size of each dog was estimated (small <10 kg, middle 11–25 kg, large 25–44 kg, or giant >45 kg breed dogs) and the dogs were categorized by predominant breed and head shape (e.g., brachycephalic). Dental intra-oral radiographs, photography, and complete oral examination were performed. Teeth with gross anomalies that could influence pulp cavity anatomy were excluded. Changes such as severe abnormal abrasion or attrition, stage 3 or 4 tooth resorption, generalized bone loss suggesting hyperparathyroidism, moderate to severe periodontal disease or complicated crown fractures were deemed significant (2, 4, 18). According to those criteria, 10 teeth from six different dogs were selected for analysis.

Following mucoperiosteal flap elevation, the MxPM4 and surrounding alveolar bone were collected en bloc, by an osteotomy through the maxilla, with a high-speed dental handpiece as described by Soltero-Rivera et al. (19). Four teeth had minimal burr cut on the outer surface of one or two apices which did not meet exclusion criteria and were kept for analysis. The samples were again photographed and radiographed. Remnants of soft tissue and adjacent teeth were removed after bathing in 2% hydrogen peroxide for 12 h. Teeth were stored at room temperature in 70% ethyl alcohol until image acquisition.

In preparation for the image acquisition, each specimen was then placed in a 50 ml polypropylene tube and covered by a storage medium (anhydrous ethanol 70%). The teeth were stabilized in the tube with plastic paraffin. They were scanned with a SkyScan 1174v2 (Bruker, Kartuizersweg 3B, 2550 Kontich, Belgium) at 50 kV source voltage and 800 μA intensity using a 1-mm-aluminum filter. Projections were recorded with 8.6 s exposure time and an angular increment of 0.5° with two acquired images per rotation on a rotation over 180°, with a spatial resolution of slices of 25 μm. The mean number of images per micro-CT study was 970.

### Images acquisition and analysis

**Figure 2:**
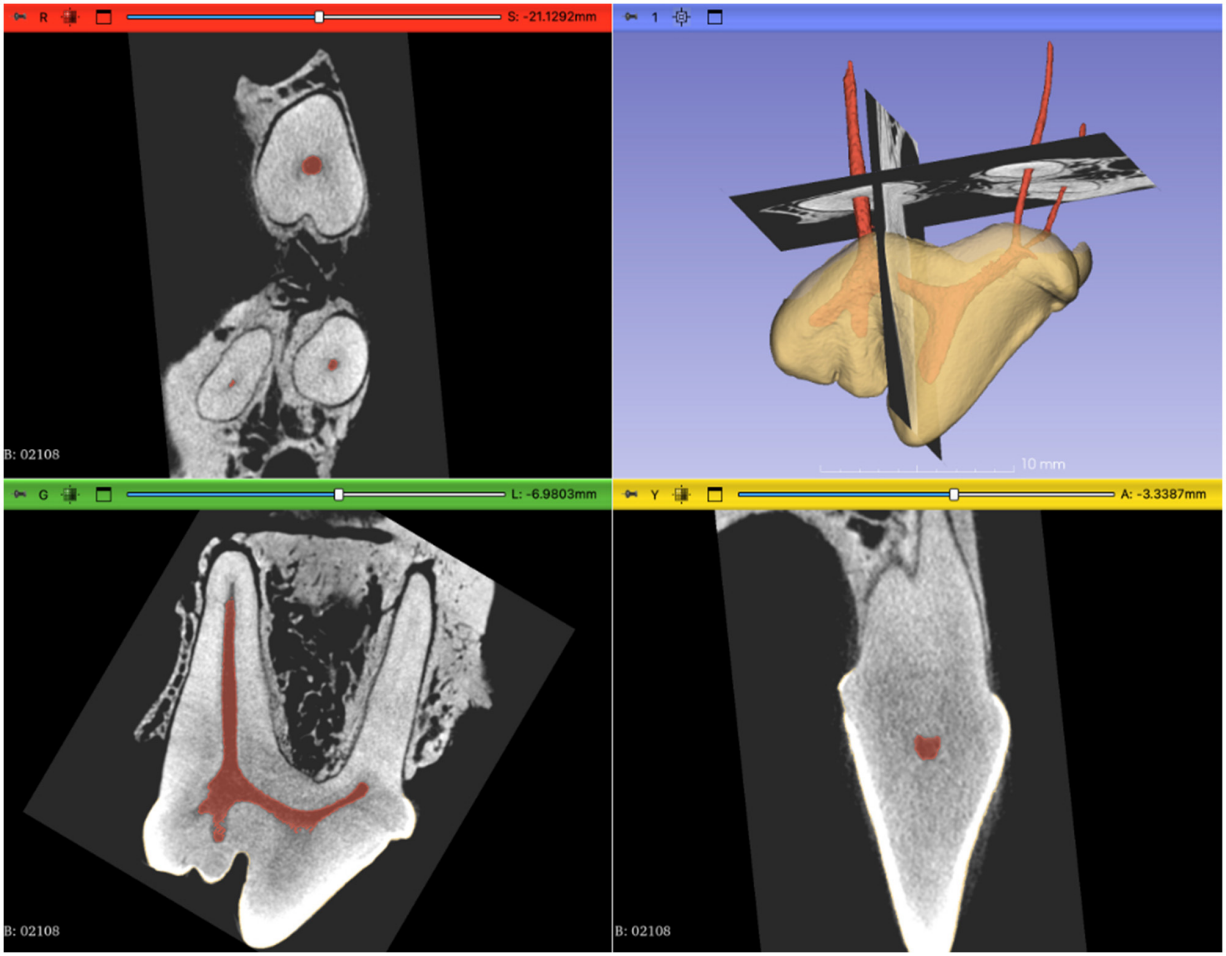
Screenshot showing the multiplanar reconstruction in coronal (red window), sagittal (green window), and axial or transverse (yellow window) view in 3D slicer. The blue window shows the 3D reconstruction with the segmentations of the crown and pulp cavity. The coronal and axial slides are shown on the 3D model. We see in the coronal view that the radicular groove did not affect the shape of the D canal. We could appreciate the fusiform shape of the D canal in the sagittal section and the arched floor and roof of the pulp chamber in the axial view.

The reconstruction of the images was carried out using the software NRecon (Bruker) and then converted into DICOM format with the software DicomCT (v2.2). A free and open-source software, 3D slicer (v5.2.1), was used to perform segmentation, reconstruction and measurements (20). Crown and pulp segmentation were done using the segmentations and segment editor modules. The threshold range was manually adjusted to select the pulp cavity or the crown. The scissors, island, and erase tools were utilized to separate the pulp cavity or the crown from interfering elements. The segmentation of dentin and roots was not performed in this study, as the alveolar bone and the dentin could not be differentiated using automated tools. Manual segmentation of the roots would have been possible but was not critical in our opinion for specimen analysis. Multiplanar reconstruction (MPR) in the axial (transverse or mesial to distal), coronal (coronal to apical) and sagittal (buccal to palatal) planes were used to verify segmentation quality and accuracy. The coronal, axial, and sagittal axis were primarily aligned with the long axis of the distal root canal and secondly with the long axis of the pulp chamber using the transforms module. The pulp and the crown were segmented for 3D reconstruction by the first author. The two authors then independently used MPR and 3D reconstruction for visualization, analysis and measurements (Figure 2). The mean between the two observer's measures was used for presenting results (Table 1). When there was no agreement for the presence of a significant angle, only one measure was recorded as indicated in Table 1.

**Table 1:** Summary of the measures on the MxPM4 canals.

	**F (°)**	**P**				**B**			**D**
		**PC1 (**°**)**	**PC2 (**°**)**	**PO-PA (mm)**	**F-PC1 (mm)**	**BC1 (**°**)**	**BC2 (**°**)**	**BO-BA (mm)**	**DO-DA (mm)**
2–108	115.7	152.4	167.9	9.6	3.4	169.5	166.8^*^	11.1	9.9
3–108	128.9	154.1	169.7^*^	7.8	3.7	159.0	NA	10.3	10.8
3–208	121.4	152.3	NA	7.3	3.4	164.2	NA	10.5	11.1
4–108	138.4	157.3	NA	7.8	3.1	171.1	NA	9.8	10.5
4–208	141.7	162.6	163.9	8.2	3.1	170.7	NA	9.8	10.6
5–108	104.3	143.1	NA	9.8	4.3	174.5	NA	10.1	10.8
5–208	109.0	147.7	170.2^*^	9.5	3.8	171.6	NA	9.8	11.3
8–108	123.2	145.7	167.3	10.0	4.3	155.8	164.2	11.4	10.7
8–208	109.5	145.4	170.6^*^	10.4	4.6	167.2	172.1^*^	10.6	10.2
9–208	106.7	144.9	NA	8.4	2.7	170.3	NA	9.0	9.9
Mean	119.8	150.5	168.3	8.9	3.6	167.4	167.4	10.2	10.6
SD	13.2	6.3	2.5	1.1	0.6	6.0	4.1	0.7	0.5

NA, No curvature.

^*^No agreement for the presence of a significant angle BC2 or PC2, only one measure was recorded.

**Figure 3:**
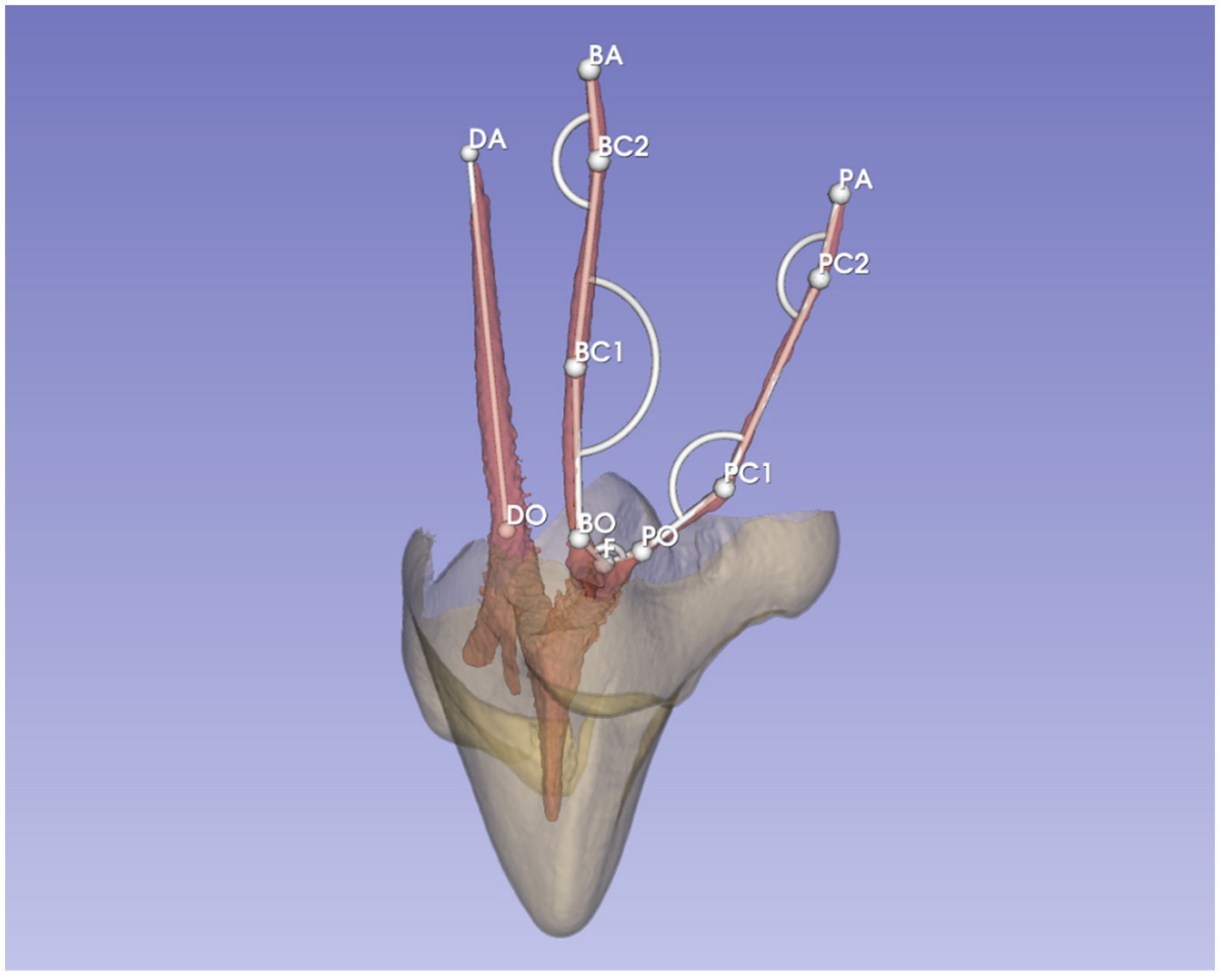
Example of marks used on specimen 2–108 for measurements of angles and length.

Different points were placed in each canal to measure angles and length (Figure 3). The markups module with the angle, curve and line tools were used. A first point (F) was placed on the floor of the pulp chamber where the two mesial canals meet. Except for this furcation point, all points were centered in the canal in the MPR views. For each canal (D, B, or P), a point was positioned at the canal orifice, located at the buccal cemento-enamel junction (CEJ) or where the funnel shape of the orifice ended (points DO, BO, or PO). The angulation between the mesial canals at the furcation was calculated by joining point PO, F, and BO (angle F). Again, for all canals, a mark was placed for each point of curvature (C) and numbered (e.g., point PC1, PC2, BC1, and BC2). The angulation of those points was calculated by joining itself with previous and following points (e.g., angle PC1 = PO-PC1-PC2). The last point was put at the end of the canal at the apex (points DA, BA, and PA). The length of the canals was calculated by joining all points with a line from the orifice up to the point at the apex. The position of PC1 in the canal was measured from point F to PC1, as this corresponds better to a clinical landmark during root canal treatment.

The apical delta and NARs were evaluated mostly using the MPR as our resolution did not allow segmentation easily. Any canal originating from the main canal and completely separated from the apical delta was considered NAR as defined by Hernandez et al. (7). Secondary canals originate from the main canal in the apical third of the root. In contrast, lateral canals emerge from the main canal in the middle or coronal third of the root.

Other findings were also noted such as tooth resorption and pulp mineralization (pulp stones). The resorptive lesions were classified using the radiographic description published by Peralta et al. (31). The shape of the canals was assessed by scrolling the MPR images in the coronal view from the orifice to the apex of each root. The shape of the distal canal was also assessed in the sagittal and axial planes. The following definitions are used in the literature to describe the shape of the canal. In a coronal view, a round canal will have a maximum diameter equal to its minimum diameter. An oval-shaped canal has a maximum cross-sectional diameter up to twice as large as its minimum diameter. A long oval-shaped canal has a cross-sectional diameter that is two to four times larger than its minimum diameter. An irregular canal has a cross-sectional shape that cannot be classified as round, oval, or long oval (3).

## Results

Two dogs (dogs 8 and 9) were estimated to be of a small breed and four were middle-sized dogs. Five dogs were mesocephalic and one was brachycephalic (dog 9). Teeth from dog 4 had proportionally larger pulp cavities than any other teeth, indicating that this dog was younger than others. Teeth were labeled with the number assigned to each dog and the modified Triadan system tooth identification number (e.g., 4–208).

**Figure 4:**
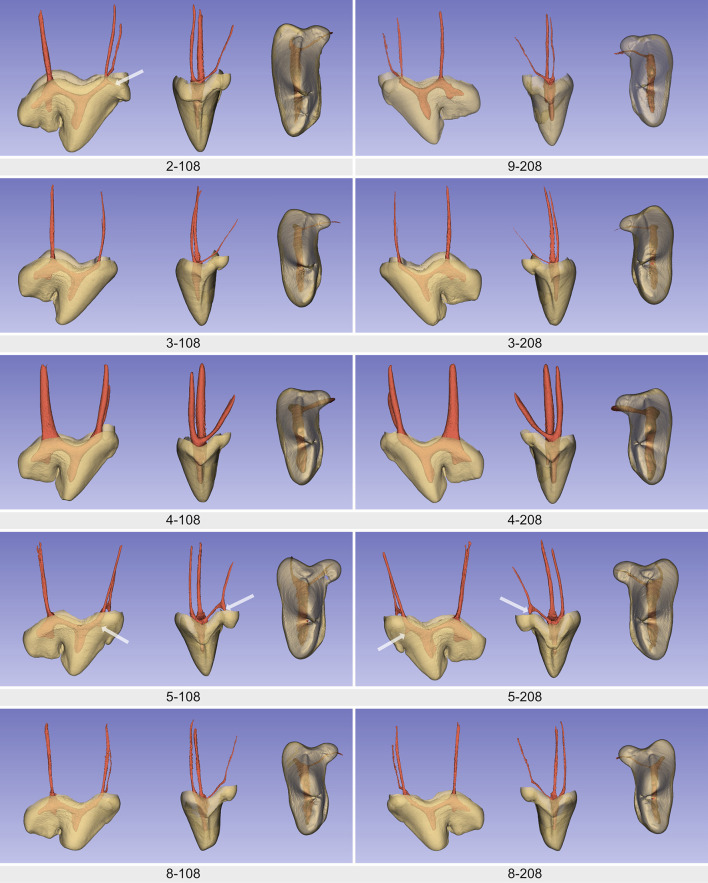
Buccal, mesial and occlusal view, respectively, of each sample's 3D reconstruction with segmentation of the crown and pulp cavity. Note that the images are at the same scale between specimens. The point of view may be slightly different. White arrows indicate accessory pulp horns related to the parastyle, the plesioconule or the protocone.

Three pulp horns were identified in all teeth and were associated with the paracone, the metacone and the metastyle (Figure 4). Overall, in the axial plane, the pulp chamber floor tended to be convex (curved inward) and the roof was arched (Figure 2). The floor of the pulp chamber in the metacone was funnel-shaped until it reaches the DO. The pulp horn of the metacone and metastyle could either be distinct (3 teeth), be partially fused (3 teeth) or completely fused together (4 teeth). Tooth 2–108 with the most prominent pulp horn in the metacone also has a notch on its edge. Smaller pulp projections were also present in the parastyle (1 tooth), the protocone (2 teeth), and the plesioconule (2 teeth; Figure 4). Specimens with these pulp projections also had the most pronounced parastyle, protocone, and/or pleisioconule of our population.

**Figure 5:**
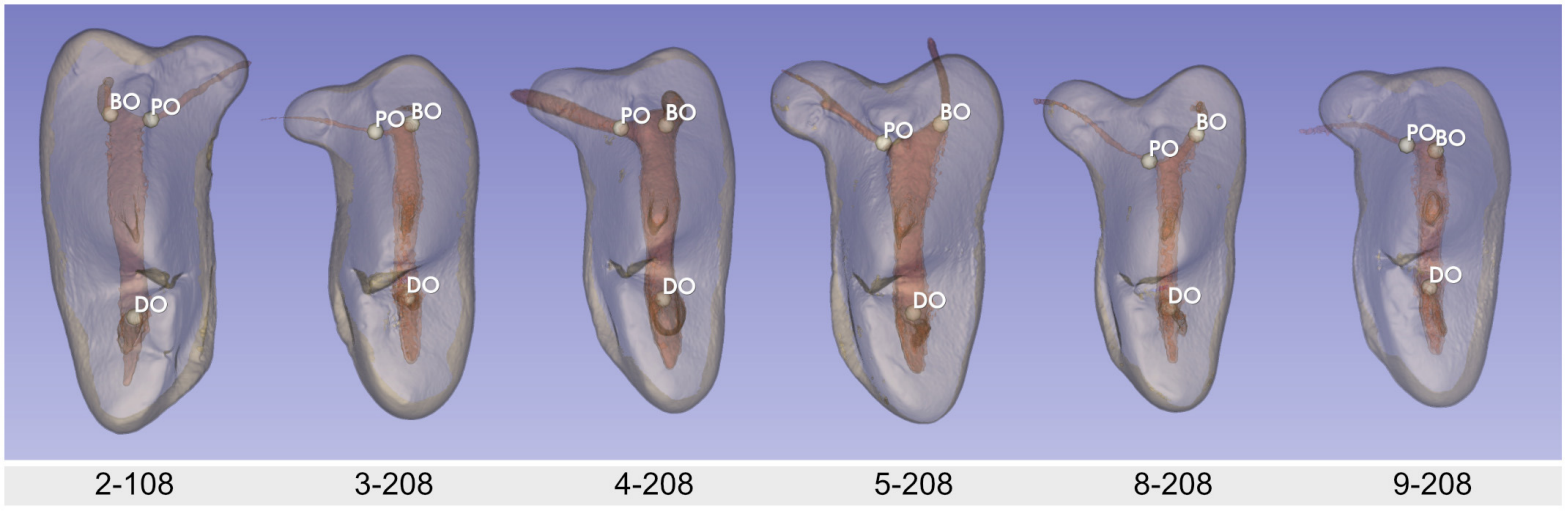
Coronal point of view of one tooth from all dogs to illustrate difference in orifice locations. BO, Buccal canal orifice; PO, Palatal canal orifice; DO, Distal canal orifice.

All teeth had three roots with a single canal, with the canal orifices located on the floor of the pulp chamber, mostly at the level of the buccal CEJ. In the coronal cross-section, the BO was slightly mesial (within a mm) or in line with the PO in 9 teeth (Figure 5). Only one tooth from the brachycephalic dog (9–208) has the PO slightly mesial to the BO. Four teeth (dog 5 and 8) exhibited a more mesiobuccally positioned BO on the coronal view. On the crowns of these teeth, a deeper mesial V-shaped cingulum was also observed (Figure 5). On the buccal view, the DO was clearly mesial (dog 9), slightly mesial (7 teeth), or aligned (2 teeth) with the metacone's pulp horn (Figure 4).

Measurments of the canals are summarized in Table 1. The first angle of the palatal canal (PC1) was obvious and present in all teeth, located below the protocone and running in a mesio-palatal direction from the PO. The line connecting PO and PC1 was parallel with the adjacent mesial crested cingulum (Figure 1). The B canals of all teeth studied had a slight curvature from the BO to the BA. A second point of curvature (BC2) was observed in only three teeth with only one having both authors' consensus. One author also noted a third angle (BC3) on two teeth (dog 8). The B canal of dog 3 was particularly curved palatally on the mesial view. The D canal was straight or slightly curved for all teeth.

The pulp canal's shape was round to oval in the coronal plane in the MDR image for the mesial roots. The B and P canals of dog 4 exhibited a fusiform shape on the three-dimensional reconstruction, whereas all the other canals showed a progressive decrease in diameter from the orifice to the apex. From its orifice to the apex, the D canal took an oval to round shape. In the sagittal plane, the D canal was fusiform for 8 teeth. Generally, the diameter of the canals were D > B ≥ P.

**Figure 6:**
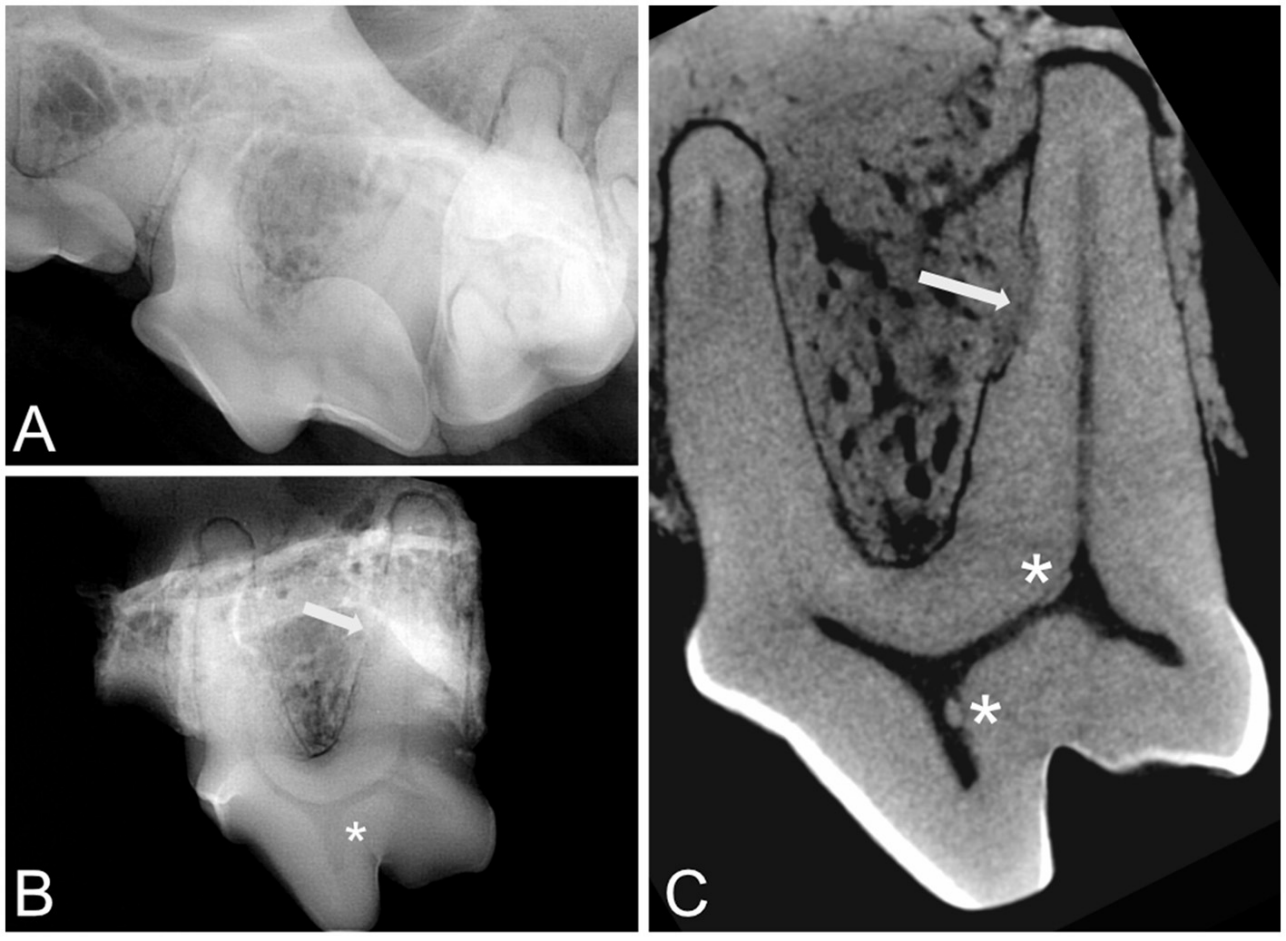
Intra-oral radiographs before **(A)** and after **(B)** sample collection for tooth 8–208. A sagittal slice **(C)** of the micro-CT shows replacement tooth resorption (white arrow) and pulp mineralizations or pulp stones (asterisk). The paracone showed abrasion that seemed to be reflected on the pulp horn shape (tertiary dentin).

Non-apical ramifications were noted in 7 teeth; all were secondary canals. At the apex of all roots, an apical delta was observed. We noted external replacement resorption or external surface resorption on 5 out of 10 teeth (Figure 6). These lesions were not associated with changes in the endodontic system. Mineralization was observed in the pulp chamber of four teeth. Three teeth exhibited mineralization in proximity to the DO (3–108, 3–208, and 8–108), while one tooth was noted to have mineralization in the pulp horn of the paracone (8–208; Figure 6). Two teeth (dog 5) had mineralization at the apex of the D canal, giving the appearance of a bifurcation.

## Discussion

Bjorndal et al. were the first to attempt to use high-resolution micro-CT to evaluate root canal anatomy in 1999. Currently, micro-CT is the most important and accurate research tool for the study of root canal anatomy (3, 21). This technique has been employed in veterinary dental studies including those investigating infundibular caries and accessory canals in horses (17, 22), malformation of carnassial teeth in dogs (14) and morphology of the pulp cavity of canine teeth in cats (13). To our knowledge, this study was the first to use this technology to evaluate the endodontic system of the Mx4PM in dogs.

We observed that the main shape of the endodontic system of MxPM4 was relatively similar for all the teeth we evaluated. We used the Osborn's terminology as it allowed us to precisely describe crown cusp variations, which was not possible with the usual veterinary or human dentistry nomenclature (2, 9, 11, 23). In our study, we have tried to establish a link between the morphology of the pulp and that of the crown. The pulp horn in the paracone showed little variation; its shape was always triangular and compressed linguo-buccally. Variations in the morphology of the metacone's pulp horn were present. The most common conformation was the complete or partial fusion with the pulp horn of the metastyle, resulting in a heart shape. The two horns may be more distinct and pronounced showing one horn in the metacone and the other in the metastyle. Projections of the pulp related to the plesioconule, the protocone or the parastyle were seen in 3 teeth.

It has been shown in humans that premolars teeth exhibit variations in the number and shape of canals. It is well-established that it can vary greatly between populations and even within the same individual (4, 24). Indeed, the tooth from the brachycephalic dog (9) showed several variations from the others. The mesial root orifices were inverted mesiodistally and the distal orifice was further mesial from the metacone's pulp horn. The breed of dog may influence the morphology of the crown, which affects the morphology of the pulp. It has been shown in foxes that variations in crown characteristics could help to distinguish one species from another (25).

Several other techniques have been used over time to study the endodontic system (3). The first study of the endodontic system in 1870 by Muhlreiter used sections of teeth to describe their internal and external morphology (3). The same technique was used to describe the endodontic system of the dog by Rochette in 1996. Regarding the MxPM4, it was found that the palatal root canal was compressed buccally to palatally such that the root canal has a long oval-shaped canal (26). In all of our teeth, this section of the canal was oval when viewed in coronal cross-section.

Another method used to study the endodontic system was to make hard tissues transparent and to fill the pulp cavity with Indian ink. Hernandez et al. employed this technique to observe dog carnassial teeth. Their observations focused on the morphology of the apical delta and the presence of NARs. Almost all NARs observed on MxPM4 were secondary canals, mainly on the distal and mesiopalatal roots (7). Similarly, we observed secondary canals in 7 teeth and apical delta in all roots. We found that 25 μm slices were not optimal for studying the ramifications of the apical delta, which had an average diameter of 32 μm for the MxPM4 in another research (6). A smaller slice thickness would be recommended for further study.

The access site on the crown for the root canal treatment should allow unrestricted passage from the coronal access point through the pulp cavity to the apex (2). Access sites have been identified and published for the dog's teeth (8, 27–29). The access point for the mesial roots as described by Eisner was effectively in close alignment with the furcation of the mesial canals in our samples (Point F). In light of our findings regarding the first curve in the mesiopalatal canal (PC1), we believe that the transcoronal approach does not allow unobstructed access to the apical third of the P root. This angle in the P canal has probably not been described before because it could not be seen on standard intra-oral radiographs, since it is in the buccal to palatal axis. Concerning the access to the distal root described by Eisner, it allows direct access to the apex via an opening on the metacone but this approach may not provide a complete exposition of the pulp horn of the metacone. Based on our observations, the alternative palatal root approach consisting of an additional access directly over the palatal root, is not complete for the instrumentation of the P canal (28, 29). The part between the furcation (point F) and the curvature (point PC1) would not be instrumented with this course. However, this technique could allow straight-line access to the apex.

Our small population with no signalment prevented us from establishing whether the variations in the endodontic system observed could be attributable to a certain breed or age of dog. In dogs, the MxPM4 must be sectioned for extraction. This particularity presents a challenge in obtaining whole and intact multirooted teeth, which limits the feasibility of conducting larger-scale studies with using micro-CT. To obtain teeth for micro-CT analysis, the alveolar bone surrounding the tooth must be harvested. On the other hand, this sampling technique has the advantage of maintaining the complete shape of the tooth and preserving details of the root margin that could be lost in a standard extraction. However, the presence of alveolar bone made the segmentation of dentin challenging with the software employed. A high level of agreement was established regarding the classification of root canal anatomy in the mandibular molars of humans between the CBCT and micro-CT scores (30). CBCT could be used to verify the observation revealed in this article with a larger population size and *in vivo*. Furthermore, CBCT may be employed to facilitate a comparative analysis of gross findings between skull types of dogs, or to monitor the evolution of pulp morphology with aging.

## Conclusion

This study of the anatomy of the MxPM4 of dogs has shown that the pulp morphology of MxPM4 was relatively similar, although variations exist in canal orifice location and pulp horn morphology and number. Meticulous observation of the crown could give us clues about pulpal variations. The presence of a curve in the mesio-palatal root, under the protocone, appeared to be the most important findings that could affect our understanding of root canal instrumentation during endodontic treatment. Similar protocols could be used to evaluate a larger population of dogs or other teeth.

## Data Availability

The raw data supporting the conclusions of this article will be made available by the authors, without undue reservation.

## References

[1] Soukup JW Hetzel S Paul A. Classification and epidemiology of traumatic dentoalveolar injuries in dogs and cats: 959 injuries in 660 patient visits (2004-2012). J Vet Dent. (2015) 32:6–14. 10.1177/089875641503200101 26197685

[2] Lobprise HB Dodd JR. Wiggs's Veterinary Dentistry: Principles and Practice. Hoboken, NJ: John Wiley & Sons (2019). p. 544.

[3] Versiani MA Basrani B Sousa-Neto A. The Root Canal Anatomy in Permanent Dentition. Cham: Springer International Publishing (2019). p. 19.

[4] Berman LH Hargreaves KM. Cohen's Pathways of the Pulp. St-Louis, MO: Elsevier (2020).

[5] Gracis M. Dental anatomy and physiology. In: BSAVA manual of canine and feline dentistry and oral surgery. Quedgeley, Gloucs: British Small Animal Veterinary Association (2018). p. 6–32.

[6] Hernández SZ Negro VB de Puch G Toriggia PG. Scanning electron microscopic evaluation of tooth root apices in the dog. J Vet Dent. (2014) 31:148–52. 10.1177/0898756414031003018195973

[7] Hernandez Negro DVM Maresca DDS. Morphologic features of the root canal system of the maxillary fourth premolar and the mandibular first molar in dogs. J Vet Dent. (2001) 18:9–13. 10.1177/089875640101800101 11968911

[8] Edward R Eisner DMV. Transcoronal approach for endodontic access to the fourth maxillary premolar in dog. J Vet Dent. (1990) 7:22–3. 10.1177/089875649000700402 2073359

[9] Dahlberg AA. International Symposium on Dental Morphology, 2nd, Royal Holloway College. Dental Morphology and Evolution. Chicago, IL: University of Chicago Press (1971). p. 350.

[10] Heymann HO Swift EJ Jr Ritter AV. Sturdevant's Art and Science of Operative Dentistry. 6th ed. t. Louis, MO: Mosby (2012). p. 568.

[11] Ungar PS. Mammal Teeth: Origin, Evolution, and Diversity. Baltimore, MD: JHU Press (2010). p. 320.

[12] Bartolini-Lucenti S Spassov N. Cave canem! The earliest Canis (Xenocyon) (Canidae, Mammalia) of Europe: taxonomic affinities and paleoecology of the fossil wild dogs. Quat Sci Rev. (2022) 276:107315. 10.1016/j.quascirev.2021.107315

[13] Chrostek E Peralta S Fiani N. Morphological study of pulp cavity anatomy of canine teeth in domestic cats using micro-computed tomography. Front Vet Sci. (2024) 11:1373517. 10.3389/fvets.2024.1373517 38523713 PMC10957770

[14] Ng KK Rine S Choi E Fiani N Porter I Fink L. Mandibular carnassial tooth malformations in 6 dogs-micro-computed tomography and histology findings. Front Vet Sci. (2019) 6:464. 10.3389/fvets.2019.00464 31956654 PMC6951429

[15] Horbal A Smith S Dixon PM. A computed tomographic and pathological study of equine cheek teeth infundibulae extracted from asymptomatic horses. Part 2: MicroCT, gross, and histological findings. Front Vet Sci. (2019) 6:125. 10.3389/fvets.2019.00125 31106214 PMC6498889

[16] De Rycke LM Boone MN Van Caelenberg AI Dierick M Van Hoorebeke L van Bree H. Micro-computed tomography of the head and dentition in cadavers of clinically normal rabbits. Am J Vet Res. (2012) 73:227–32. 10.2460/ajvr.73.2.227 22280382

[17] Korsós SA Staszyk C Boone M Josipovic I Vogelsberg J Vlaminck L. Micro-CT and histological examination of accessory canals in 34 equine cheek teeth. Front Vet Sci. (2024) 11:1396871. 10.3389/fvets.2024.1396871 38659446 PMC11039908

[18] Gautam S Galgali SR Sheethal HS Priya NS. Pulpal changes associated with advanced periodontal disease: a histopathological study. J Oral Maxillofac Pathol. (2017) 21:58–63. 10.4103/0973-029X.203795 28479688 PMC5406820

[19] Soltero-Rivera M Elliott MI Hast MW Shetye SS Castejon-Gonzalez AC Villamizar-Martinez LA. Fracture limits of maxillary fourth premolar teeth in domestic dogs under applied forces. Front Vet Sci. (2018) 5:339. 10.3389/fvets.2018.00339 30761310 PMC6364561

[20] Fedorov A Beichel R Kalpathy-Cramer J Finet J Fillion-Robin J-C Pujol S. 3D Slicer as an image computing platform for the Quantitative Imaging Network. Magn Reson Imaging. (2012) 30:1323–41. 10.1016/j.mri.2012.05.001 22770690 PMC3466397

[21] Bjørndal L Carlsen O Thuesen G Darvann T Kreiborg S. External and internal macromorphology in 3D-reconstructed maxillary molars using computerized X-ray microtomography. Int Endod J. (1999) 32:3–9. 10.1046/j.1365-2591.1999.00172.x 10356463

[22] Horbal A Smith S Dixon PM. A computed tomographic (CT) and pathological study of equine cheek teeth infundibulae extracted from asymptomatic horses. Part 1: prevalence, type and location of infundibular lesions on CT imaging. Front Vet Sci. (2019) 6:124. 10.3389/fvets.2019.00124 31106213 PMC6494954

[23] Berkovitz BKB Shellis P. The Teeth of Mammalian Vertebrates. Cambridge, MA: Academic Press (2018). p. 346.

[24] Ahmed HMA Versiani MA De-Deus G Dummer PMH. A new system for classifying root and root canal morphology. Int Endod J. (2017) 50:761–70. 10.1111/iej.12685 27578418

[25] Gimranov DO. Structure of the upper teeth of the red fox (*Vulpes vulpes*) and Arctic fox (*Vulpes lagopus*) and analysis of dental variability in insular forms. Russ J Theriol. (2021) 20:96–110. 10.15298/rusjtheriol.20.1.10

[26] Rochette J. ldentification of the endodontic system in carnassial and canine teeth in the dog. J Vet Dent. (1996) 13:35–7. 10.1177/089875649601300104

[27] Visser CJ. Coronal access of the canine dentition. J Vet Dent. (1991) 8:12–5. 10.1177/089875649100800403 1815631

[28] Harvey CE Emily P. Small Animal Dentistry. Baltimore, MD: Mosby (1993). p. 413.

[29] Holmstrom SE Frost P Eisner ER. Veterinary Dental Techniques: For the Small Animal Practitioner. Philadelphia, PA: Saunders (2004). p. 689.

[30] Pires M Martins JNR Pereira MR Vasconcelos I Costa RPd Duarte I. Diagnostic value of cone beam computed tomography for root canal morphology assessment—a micro-CT based comparison. Clin Oral Investig. (2024) 28:201. 10.1007/s00784-024-05580-y 38453706 PMC10920457

[31] Peralta S Verstraete FJ Kass PH. Radiographic evaluation of the classification of the extent of tooth resorption in dogs. Am J Vet Res. (2010) 71:7948. 10.2460/ajvr.71.7.794 20594082

